# A Portable Personal Glucometer-Based Immunoassay Platform for Horseradish Peroxidase-Related Biomarker Detection

**DOI:** 10.3390/bios16090522

**Published:** 2026-09-19

**Authors:** Jia-Yuan He, Ming-Shan Shuai, Hao Zhang, Dan-Ni Yang

**Affiliations:** 1 Traditional Chinese Medicine College, Chongqing University of Chinese Medicine, Chongqing 402760, China; 2 The Central Laboratory, The Affiliated Dazu’s Hospital of Chongqing Medical University, Chongqing 402360, China; 3 Chongqing Engineering Research Center of Pharmaceutical Sciences, Chongqing Medical and Pharmaceutical College, Chongqing 401331, China

**Keywords:** personal glucometer, horseradish peroxidase, immunoassay, biomarker detection

## Abstract

Given the prevalent use of horseradish peroxidase (HRP) as an enzyme label in immunoassays, the practical importance of point-of-care testing for the portable identification of HRP-related biomarkers is evident. Accordingly, this study devised a portable immunoassay platform using a personal glucometer (PGM) to achieve rapid detection of HRP-related biomarkers. 3,3′,5,5′-tetramethylbenzidine (TMB) and dopamine (DA) were used as signal-regulating substrates, and the assay principle was based on the HRP-mediated redox reaction. In the absence of HRP, TMB and DA directly reacted with [Fe(CN)_6_]^3−^ on commercial glucose test strips, producing a strong PGM response. When HRP and H_2_O_2_ were present, HRP facilitated the oxidation of TMB to generate oxidized TMB, which further reacted with DA to produce oxidized products with weak reactivity toward [Fe(CN)_6_]^3−^, resulting in a decreased PGM signal. Conducting the quantitative assay involved measuring the HRP-dependent change in the PGM readout. Unlike many PGM immunoassays that rely on additional glucose-producing enzyme labels or amplified nanomaterial tags, the proposed method directly converts the signal of the conventional HRP label into a PGM-readable response. The sensing platform demonstrated a good linear response to HRP concentrations ranging from 156 to 625 ng/mL, with a low detection limit of 26 ng/mL. Interference experiments indicated good tolerance to the tested nonspecific proteins. The preliminary applicability of the platform was further evaluated by detecting cardiac troponin I (cTnI) and heparin-binding protein (HBP) in spiked serum samples, with recoveries ranging from 93.0% to 117.1% for cTnI and from 88.3% to 112.5% for HBP. In summary, the proposed platform provides a portable digital readout for HRP-linked immunoassays and reduces the dependence on dedicated readout instruments.

## 1. Introduction

Horseradish peroxidase (HRP) is an oxidoreductase that depends on heme and is extracted from the roots of horseradish. It has been widely adopted as a standard enzyme marker in the field of bioanalytical chemistry. In the presence of H_2_O_2_, this enzyme can mediate the one-electron oxidation process of various hydrogen donors, thereby generating colored, fluorescent or chemiluminescent products. Common substrates include 3,3′,5,5′-tetramethylbenzidine (TMB), *o*-phenylenediamine, 2,2′-azino-bis(3-ethylbenzothiazoline-6-sulfonic acid), luminol, phenolic compounds and aniline derivatives [1]. Due to the strong catalytic activity, excellent operational stability, convenient bioconjugation and wide compatibility with various signal output responses of HRP, this enzyme has been widely applied in clinical testing, food safety monitoring, environmental analysis, biosensing and enzyme-linked immunosorbent assay [2,3]. In most analytical systems, HRP, as a signal amplification element, can convert the target recognition process into measurable physical and chemical signals.

To assess the activity of HRP, various analytical strategies have been developed, including colorimetry [4], fluorescence [5], chemiluminescence [6], and surface-enhanced Raman scattering [7]. Among these, colorimetry is favored due to its low cost, simplicity of operation, and suitability for detection through visual observation or absorbance measurement; however, its analytical sensitivity may be limited by factors such as weak color contrast, sample matrix background interference, and the limited signal gain produced by traditional chromogenic substrates [4]. Fluorescence-based detection methods usually have high sensitivity and fast response speed, but many reported designs rely on specifically synthesized probes or indirect signal regulation mechanisms (such as fluorescence quenching mediated by the internal filter effect), which may increase the complexity of the detection system construction and affect background correction [5]. Chemiluminescence systems can achieve high sensitivity detection without an external excitation light source, but they usually require dedicated luminescence detection instruments and precise control of the reaction time [6]. Surface-enhanced Raman scattering methods can further enhance detection sensitivity, but the reproducibility problem in the preparation of the enhancement substrate and the dependence on Raman spectroscopy instruments may limit their wide application in routine analysis [7]. Therefore, despite the progress of HRP assays, a portable, low-cost, and broadly adaptable HRP readout strategy is still needed for point-of-care bioanalysis.

Enzyme-linked immunosorbent assay (ELISA) is a classic immunoanalytical method utilized for quantifying proteins and other biomarkers, and HRP is one of the most frequently used reporter enzymes in this format [8,9]. In a typical HRP-linked assay, HRP is coupled to antibodies, streptavidin, or other recognition probes, and the amount of bound enzyme reflects the concentration of the target biomarker. HRP-mediated ELISA and related methodologies have been employed in the identification of cardiac troponin I, SARS-CoV-2 nucleocapsid protein, tumor extracellular vesicles, exosome-associated biomarkers, and other clinically relevant targets [2,10]. However, conventional HRP-based immunoassays usually depend on microplate readers, fluorescence spectrometers, chemiluminescence analyzers, or other laboratory instruments, which weakens their applicability in resource-limited or on-site settings. Personal glucometer (PGM), originally commercialized for blood glucose monitoring, have gradually been repurposed as portable quantitative readout devices for non-glucose targets because they are inexpensive, compact, user-friendly, and capable of producing reliable digital signals [11,12]. By coupling target recognition with glucose-producing or glucose-consuming reactions, PGM-based assays have been extended to DNA, proteins, small-molecule toxins, enzyme activities, and disease-related biomarkers [11,12,13,14]. In particular, several PGM-based immunoassays have been reported for neuron-specific enolase, tumor markers, *Cronobacter sakazakii*, and alpha-fetoprotein [15,16,17,18,19,20,21,22]. These methods employed glucose-generating enzyme labels, nanomaterial-assisted amplification, rolling-circle amplification, or electrochemical signal-conversion strategies to produce PGM-readable signals. These previous studies established the feasibility of PGM-based immunoanalysis. However, many reported systems require the preparation of additional glucose-producing enzyme labels, antibody-nanomaterial conjugates, DNA-amplification components, or specialized electrochemical sensing interfaces. In contrast, HRP is already one of the most commonly used enzyme in conventional ELISA kits. Therefore, direct conversion of the signal from an HRP label into a PGM-readable response would provide a practical route for adapting conventional HRP-linked immunoassays to portable digital analysis. Thus, integrating HRP-linked immunoreactions with a PGM readout provides a promising route for constructing a universal and portable immunoassay platform for HRP-related biomarker detection.

**Figure 1:**
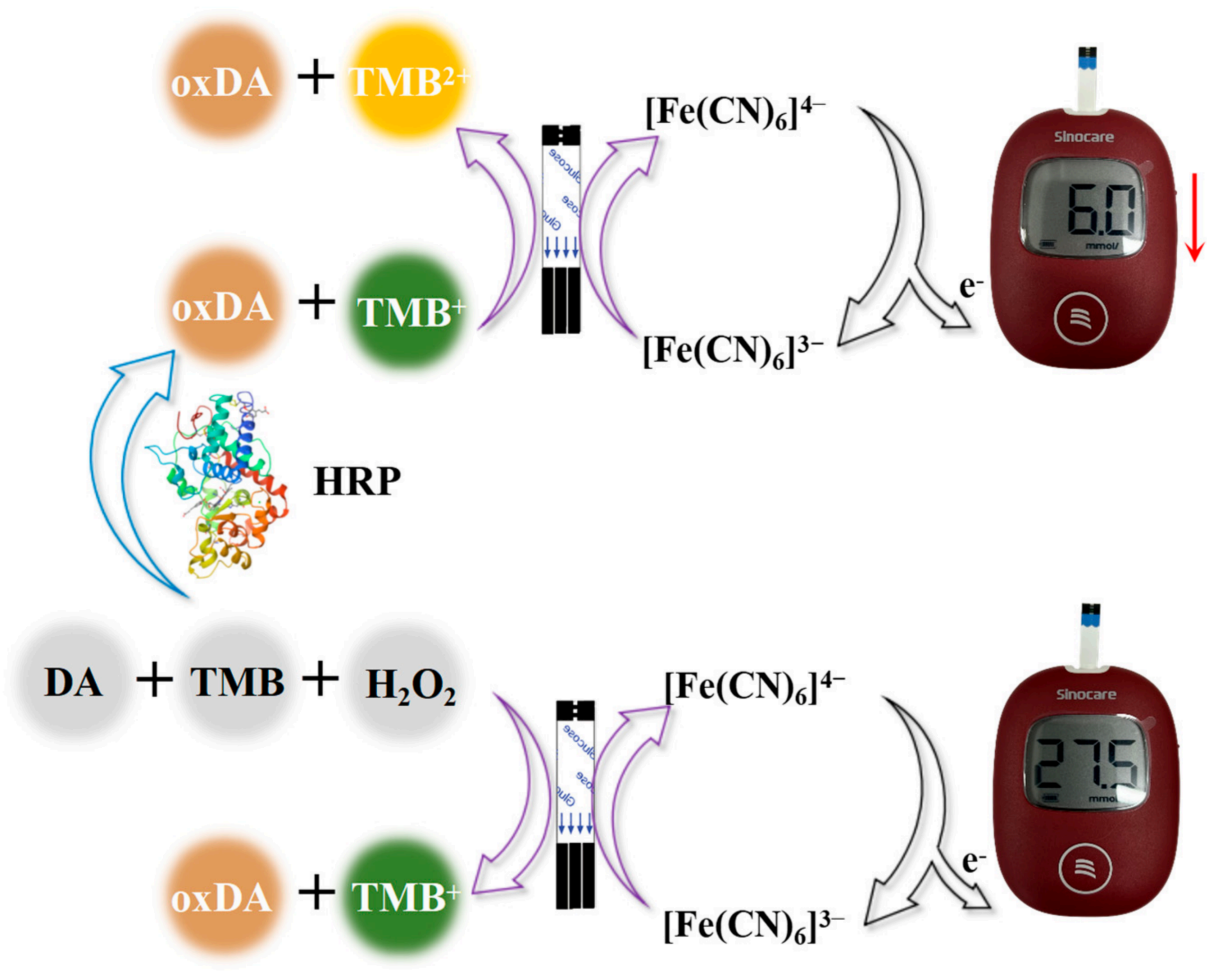
Schematic illustration of the detection principle of the personal glucometer method for HRP activity based on the DA/TMB/H_2_O_2_-mediated signal conversion process.

In this study, a portable PGM-based immunoassay platform was established to detect HRP-related biomarkers. This method combines the redox signal mediated by HRP with the advantages of PGM for precise quantitative assessment. In this system, HRP serves as the key signal label in the immunoreaction, while TMB and dopamine (DA) act as electroactive mediators to regulate the PGM response. In the absence of HRP, TMB and DA can directly react with [Fe(CN)_6_]^3−^ on the glucose test strip, leading to a strong PGM signal. When HRP and H_2_O_2_ are present, HRP facilitates the oxidation of TMB to generate oxidized TMB, which further reacts with DA to produce oxidized products with weak reactivity toward [Fe(CN)_6_]^3−^ (Figure 1). The principal contribution of this work is not the achievement of the lowest detection limit (LOD), but the direct translation of a conventional HRP label into a commercial PGM readout without introducing an additional glucose-producing enzyme label or a complex amplification tag. The key experimental conditions, including pH value, temperature, the concentrations of H_2_O_2_, TMB, DA, and the enzymatic reaction time, have all been optimized. Subsequently, the established PGM-based analytical method was used for the determination of HRP. To further demonstrate the applicability of the PGM immunological analysis platform, the platform was subsequently applied to biomarker detection in spiked serum sample, demonstrating its potential for point-of-care-oriented biomarker analysis.

## 2. Materials and Methods

### 2.1. Chemicals and Materials

Hydrogen peroxide and absolute ethyl alcohol were obtained from Chengdu Kelong Chemical Co., Ltd. (Chengdu, China). HRP, α-amylase, trypsin and β-glucosidase were obtained from Shanghai Yuanye Biological Technology Co., Ltd. (Shanghai, China). TMB, lysozyme and bovine serum albumin were purchased from Shanghai Titan Scientific Co., Ltd. (Shanghai, China). Collagen was obtained from Shanghai Macklin Biochemical Technology Co., Ltd. (Shanghai, China). Human heparin-binding protein (HBP) ELISA kit was purchased from Hangzhou Zhenyoupin Biotechnology Co., Ltd. (Hangzhou, China). Human serum was purchased from Beijing Solarbio Science and Technology Co., Ltd. (Beijing, China). DA was acquired from Shanghai Aladdin Biochemical Technology Co., Ltd. (Shanghai, China). Human Cardiac troponin I (cTnI)/TNNI3 ELISA kit was purchased from Shanghai Sangon Biotech Co., Ltd. (Shanghai, China).

### 2.2. Instrumentation

The PGM (Sannuo+) and glucose test strips were acquired from Sinocare Inc. (Changsha, China). A DHG-9035A drying oven was acquired from Shanghai Yiheng Technology Instrument Co., Ltd. (Shanghai, China), while a Vortex mixer XW-80A type was obtained from Kylin-Bell Lab Instruments Co., Ltd. (Nantong, China), and the SB-4200DT ultrasonic cleaner was purchased from Ningbo Scientz Biotechnology Co., Ltd. (Ningbo, China). Deionized water is prepared by using the BLH1-10L-AD ultra-pure water machine of Chongqing Huachuang Water Treatment Engineering Co., Ltd. (Chongqing, China).

### 2.3. Preparation of Solutions

HRP (1.0 mg/mL), H_2_O_2_ (10.0 mM), and DA (6.5 mg/mL) were each dissolved in 10.0 mM sodium acetate buffer (pH 7.0) to prepare the respective solutions, while TMB (6.0 mg/mL) was dissolved in an ethanol solution. The interfering substances used include lysozyme, albumin, collagen, β-glucosidase, α-amylase and trypsin. All of them were prepared in a 10.0 mM sodium acetate buffer solution (pH 7.0). The solutions in the kit were prepared according to the relevant instructions.

### 2.4. HRP Analysis

First, mix 2.0 µL HRP with 2.0 µL H_2_O_2_. Then, add 2.0 µL TMB solution and DA solution respectively to the mixture, and incubate the resulting solution at 40 °C for 5.0 min. Next, take 2.0 µL of the reaction product for PGM measurement. Use the solution without HRP as the control group, and follow the above steps for the remaining operations. The analysis process is carried out in a step-by-step sequence. Each sample is mixed with the reaction reagent, and the timer for each sample is started immediately after the reagent is added. When one sample is in the incubation stage, the next sample is prepared and its reaction start time is independently recorded. All reaction mixtures were immediately transferred to commercial glucose test strips for PGM measurement after exactly 5.0 min of incubation, ensuring that the interval between reagent addition and PGM readout was independently controlled at 5.0 min for each sample. The difference in PGM response was defined as ΔP = P_Control_ − P_HRP_, where P_Control_ denotes the signal from the HRP-free control and P_HRP_ represents the signal obtained from the HRP-containing system. To evaluate the anti-interference capability of the proposed PGM-based assay for HRP detection, lysozyme, albumin, collagen, β-glucosidase, α-amylase, and trypsin were selected as possible interfering species. For the interference assay, HRP was mixed with each interfering substance, followed by PGM measurement according to the established procedure. The control group consisted of samples containing only HRP. The final concentrations of HRP and each interfering substance were both set at 1250 ng/mL.

### 2.5. Immunoassay for cTnI and HBP Based on PGM

The solution was prepared according to the instructions provided in the kit. The detection method for cTnI is as follows: Take a 96-well plate, add 50.0 μL of standard working solution or the test sample into each reaction well, seal the plate, and incubate it in a 37 °C incubator for 10.0 min. Discard the liquid from the reaction wells and allow them to dry. Then add 100.0 μL of biotin-labeled cTnI antibody working solution to each well, seal the plate, and incubate at 37 °C for 10.0 min. Next, dispose of the liquid, desiccate it, and introduce 200.0 μL of washing solution. Following this step, apply 100.0 μL of HRP-labeled streptavidin working solution to each reaction well, seal the plate, and incubate it in a 37 °C incubator for 10.0 min. Then, add 200.0 μL of washing solution, repeat twice. Finally, add 25.0 μL of H_2_O_2_, 25.0 μL of TMB, and 25.0 μL of DA to each reaction well, seal the plate, and allow it to color under light protection at 37 °C for 10.0 min. Then, 2.0 μL of the reaction mixture was taken and measured using a portable PGM. The detection method of HBP is as follows: Combine 50.0 μL of the standard working solution or the test sample with 100.0 μL of the biotin-labeled HBP antibody working solution in each reaction well of a 96-well plate. Following incubation of the sealed plate at 37 °C for 15.0 min, discard the liquid, dry the plate, and then add 200.0 μL of washing solution to each reaction well. Repeat this process twice. In each reaction well, introduce 100.0 μL of HRP-labeled streptavidin working solution, seal the plate, and then incubate at 37 °C for 15.0 min. Subsequently, add 200.0 μL of washing solution to each reaction well and repeat this process twice. Finally, add 25.0 μL of 10.0 mM acetate buffer (pH = 7.0), 25.0 μL H_2_O_2_, 25.0 uL TMB and 25.0 uL DA to each reaction well. Seal the plate and allow it to develop color in the dark for 15.0 min at 37 °C. Take 2.0 uL of the reaction mixture and measure it using a personal glucometer. For the interference assay, cTnI or HBP was mixed with each interfering substance, followed by PGM measurement according to the established procedure. Both cTnI and each interferent had final concentrations of 25.0 ng/mL, as did HBP and each interferent at 5.0 ng/mL.

## 3. Results and Discussion

### 3.1. Principle of the PGM Assay

The sensing principle is illustrated in Figure 1. Both DA and TMB can reduce [Fe(CN)_6_]^3−^ on commercial glucose test strips to [Fe(CN)_6_]^4−^, thereby producing a strong PGM response. In the DA+TMB+H_2_O_2_ system, following the introduction of HRP, TMB undergoes oxidation catalyzed by HRP to yield the blue oxidized product TMB^+^. Meanwhile, DA can reduce TMB^+^ back to TMB, accompanied by the formation of oxidized DA. These oxidation products show only weak responses on the glucose strips, resulting in a decreased PGM readout. Based on this signal-changing mechanism, rapid and portable detection of HRP can be achieved using a PGM.

**Figure 2:**
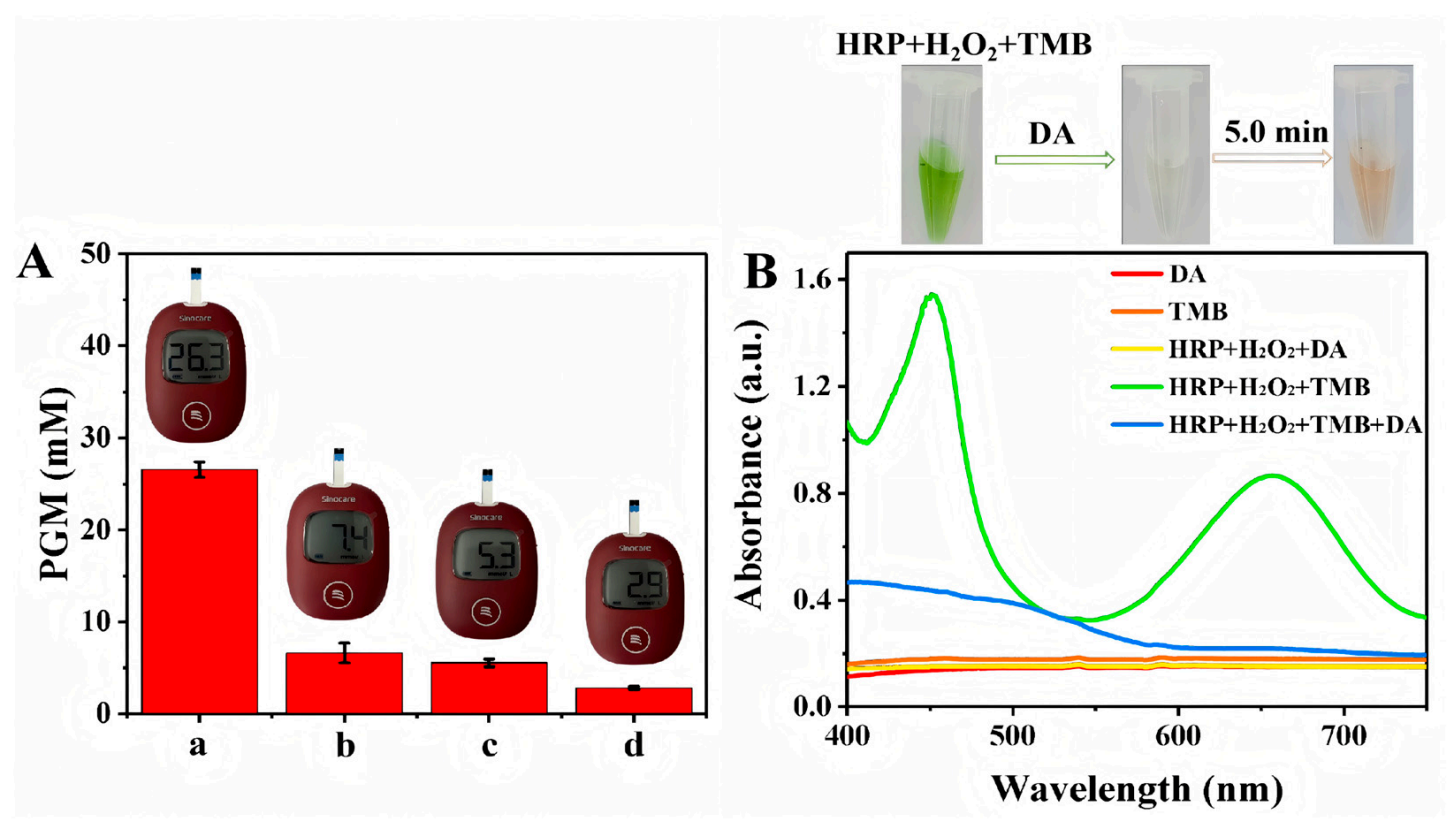
(**A**) The PGM readout of four solutions. a: DA+TMB+H_2_O_2_ solution; b: HRP+DA+TMB+H_2_O_2_ solution; c: HRP+DA+H_2_O_2_ solution; d: HRP+TMB+H_2_O_2_. (**B**) UV–vis spectra of different solutions.

In Figure 2A, the system without HRP produced the maximum PGM response (a), mainly because TMB and DA could directly react with potassium ferricyanide loaded on the glucose test strip. However, the PGM signal decreased significantly after HRP was added to the reaction system (b). The observed result is explicable through the HRP-catalyzed TMB oxidation in the presence of H_2_O_2_, followed by the reaction between oxidized TMB and DA. Consequently, oxidized TMB and oxidized DA were generated as the final products, both of which showed low responses on the PGM. Moreover, the HRP+H_2_O_2_+TMB and HRP+H_2_O_2_+DA systems also gave low PGM readouts, supporting the conclusion that HRP-mediated oxidation suppresses the PGM signal by consuming electroactive TMB and DA.

Figure 2B illustrates the UV-vis absorption spectra recorded for different reaction mixtures. The wavelength range of 400–750 nm did not exhibit any evident absorption peak for the DA or TMB solution. Similarly, the HRP+H_2_O_2_+DA solution showed no characteristic absorption peak, indicating that DA could not be efficiently oxidized by HRP under the tested conditions. In contrast, two strong absorption peaks appeared at approximately 450 nm and 650 nm for the HRP+H_2_O_2_+TMB solution, which were ascribed to the production of oxidized TMB resulting from HRP-catalyzed TMB oxidation in the presence of H_2_O_2_. After DA was introduced into the HRP+H_2_O_2_+TMB solution, the blue hue of oxidized TMB faded because DA reduced oxidized TMB back to TMB, while DA itself was oxidized to brown oxidized DA. Consequently, an extensive absorption band was observed in the 400–600 nm range, leading to the disappearance of the absorption peak at 650 nm. These spectral changes confirm the reaction between oxidized TMB and DA, supporting the proposed PGM signal-regulation mechanism. These results further demonstrate that HRP-mediated TMB oxidation and the subsequent redox reaction between oxidized TMB and DA are responsible for the decreased PGM response observed in Figure 2A.

### 3.2. Optimization of Reaction Parameters

**Figure 3:**
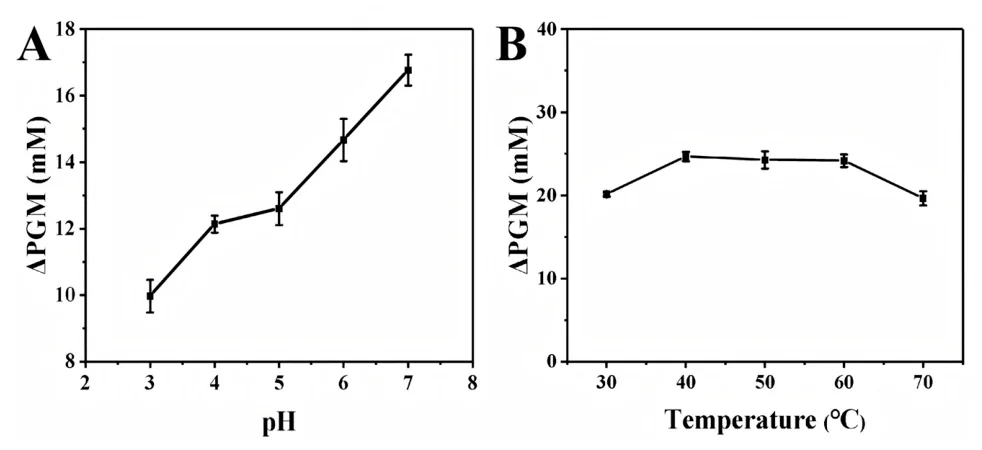
Effect of the pH (**A**) and the enzymatic reaction temperature (**B**) on the increment of the glucometer readout.

The developed HRP detection strategy consists of an HRP-driven oxidation process and a DA-dependent PGM signal response. To ensure the reliability of the analytical performance, several key experimental parameters were systematically optimized, including the reaction pH value (3.0–7.0), incubation temperature (30–70 °C), H_2_O_2_ concentration (0.125–5.0 mM), TMB concentration (0.5–2.5 mg/mL), DA concentration (0.0625–0.8125 mg/mL), and catalytic reaction time (1.0–9.0 min), on the ΔPGM value. Meanwhile, a mixture without HRP was used as a blank control. To optimize the reaction conditions of the HRP-mediated system, the influence of pH on the signal of the PGM was first investigated. Figure 3A shows that as the pH gradually increased from 3.0 to 7.0, the ΔPGM value continued to rise, indicating that under near-neutral conditions, the oxidation reaction catalyzed by HRP and the resulting DA-related signal response of the glucometer are more favorable. At the same time, in weakly alkaline conditions, DA is prone to self-polymerization, which may have an adverse effect on the operational stability of the detection method. Based on a comprehensive assessment of the signal response effect and experimental operability, the neutral condition (pH 7.0) was ultimately determined to be adopted. In addition, the effect of temperature on the ΔPGM value was also evaluated. Figure 3B shows that as the temperature increased from 30 °C to 40 °C, the ΔPGM value increased accordingly; within the range of 40 °C to 60 °C, the ΔPGM value remained relatively stable, forming a plateau; however, when the temperature reached 70 °C, the ΔPGM value significantly decreased. This trend may be related to the improved catalytic efficiency of HRP at moderate temperatures and the partial enzyme inactivation caused by high temperatures. Based on the comprehensive consideration of signal intensity and the mildness of reaction conditions, the subsequent experiments determined 40 °C as the optimal reaction temperature.

**Figure 4:**
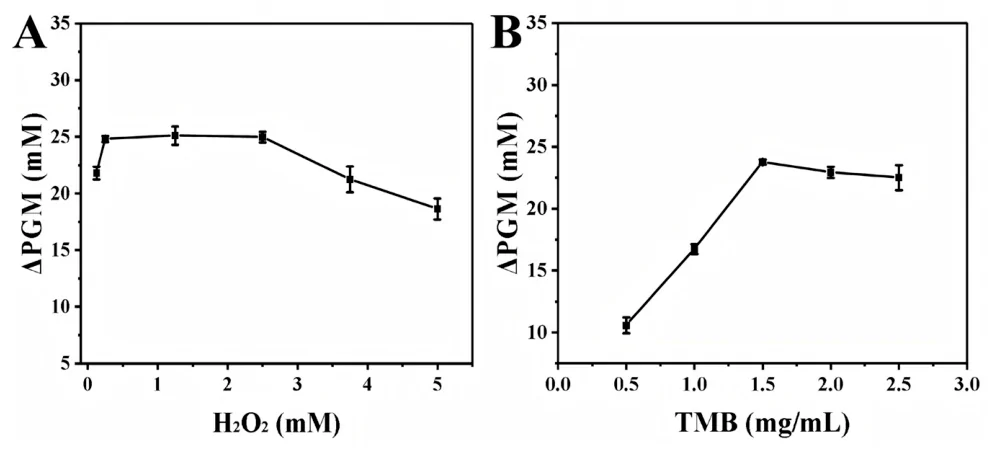
Effect of H_2_O_2_ concentration (**A**) and TMB concentration (**B**) on the increment of the glucometer readout.

Figure 4A shows the effect of H_2_O_2_ concentration changes on the ΔPGM value. When the H_2_O_2_ concentration increased from 0.125 mM to 0.25 mM, the signal showed a slight enhancement; then it remained relatively stable within the range of 0.25–2.5 mM. As the concentration continued to increase, the ΔPGM value significantly decreased. It is speculated that this is due to the excessive H_2_O_2_ inhibiting the activity of HRP or interfering with the oxidation reaction process, thereby weakening the efficiency of the reaction. Based on this, the subsequent experiments used a H_2_O_2_ concentration of 0.125 mM. Subsequently, the influence of different TMB concentrations on the ΔPGM value was investigated. In Figure 4B, when the TMB concentration increased from 0.5 mg/mL to 1.5 mg/mL, the ΔPGM value significantly rose, indicating that sufficient TMB effectively promoted the oxidation reaction catalyzed by HRP. Further increasing the TMB concentration, the ΔPGM value only showed slight changes and tended to stabilize, suggesting that the reaction system was approaching saturation. Therefore, in the subsequent experiments, a TMB concentration of 1.5 mg/mL was selected.

**Figure 5:**
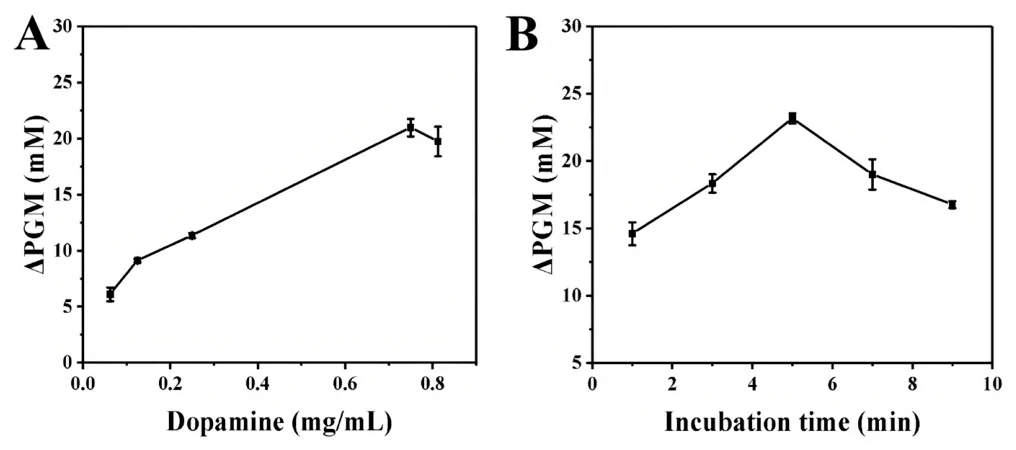
Effect of dopamine concentration (**A**) and incubation time (**B**) on the increment of the glucometer readout.

The relationship between DA concentration and ΔPGM value was further analyzed. In Figure 5A, when the DA concentration gradually increased from 0.0625 mg/mL to 0.75 mg/mL, the ΔPGM value continued to rise, indicating that a higher concentration of DA could enhance the signal difference detected by the PGM in the HRP catalytic system. However, when the DA concentration was further increased to 0.812 mg/mL, the ΔPGM value slightly decreased, which might be attributed to the enhanced background response caused by excessive DA or interference with the reaction balance. Therefore, in the subsequent experiments, the optimal concentration of DA was determined to be 0.75 mg/mL, and the incubation time was further optimized to improve the analytical performance of the HRP-mediated PGM detection method. Figure 5B shows that when the incubation time was extended from 1.0 min to 5.0 min, the ΔPGM value showed a gradually increasing trend, indicating that prolonging the reaction time can enhance the oxidation process mediated by HRP and accelerate the consumption of DA. However, if the incubation time exceeded 5.0 min, the ΔPGM value significantly decreased. This phenomenon may be related to the instability of the oxidation intermediate products or the side reactions caused by prolonged incubation. Based on the above results, the subsequent experiments adopted 5.0 min as the incubation time. Based on the above optimization results, the optimized conditions for the HRP detection were selected as follows: pH 7.0, 40 °C, 0.125 mM H_2_O_2_, 1.5 mg/mL TMB, 0.75 mg/mL DA, and a 5.0 min reaction time.

### 3.3. Analytical Performance

**Figure 6:**
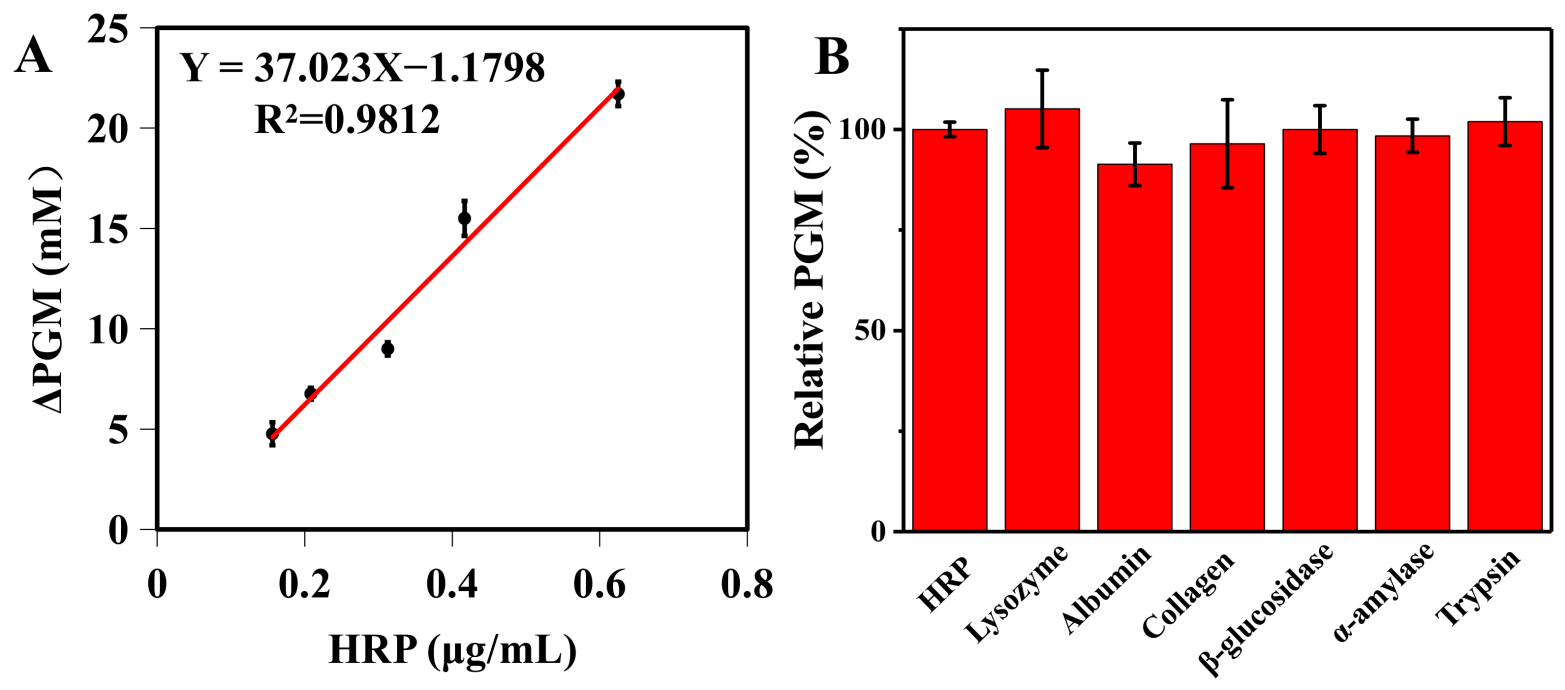
(**A**) Calibration plot for quantitative determination of HRP and interference study (**B**) using the proposed personal glucometer assay.

The repeatability of the proposed HRP detection method was evaluated using the relative standard deviation (RSD%) of the glucometer signal. The results of three repeated experiments showed an RSD of 4.7%, indicating that the method has good repeatability. On this basis, a calibration curve was established using HRP concentration as the independent variable and ΔPGM as the response signal. Figure 6A indicates that within the test range, the response signal shows a significant concentration dependence with changes in HRP concentration. The fitted regression was Y = 37.023X − 1.1798, with R^2^ = 0.9812. The results indicate that this method exhibits a good linear relationship within the concentration range of 156 to 625 ng/mL, with an LOD of 26 ng/mL. These results suggest that the strategy based on the PGM is applicable for quantitative analysis of HRP and has satisfactory sensitivity. The analytical performance of the proposed method should be interpreted in the context of portable PGM-based immunoassays rather than as a direct competitor to amplification-intensive laboratory HRP assays. Although certain laboratory methods provide lower LOD (Table 1), they commonly require optical instruments, electrochemical workstations, or specialized amplification materials. The present assay instead uses a commercial PGM and disposable glucose strips to read the signal from a conventional HRP label, which offers practical advantages in portability and assay compatibility. Figure 6B shows that in the mixed samples containing HRP and different interfering substances, the relative PGM response value is still close to the control group containing only HRP. Among the tested interferents, albumin caused the greatest deviation from control values, while other proteins and enzymes induced relatively minor changes. Despite this difference, none of the tested interferents significantly affected the HRP-dependent PGM signal, indicating that the method is highly tolerant to the studied interferents.

### 3.4. PGM-Based Detection of cTnI and HBP

**Figure 7:**
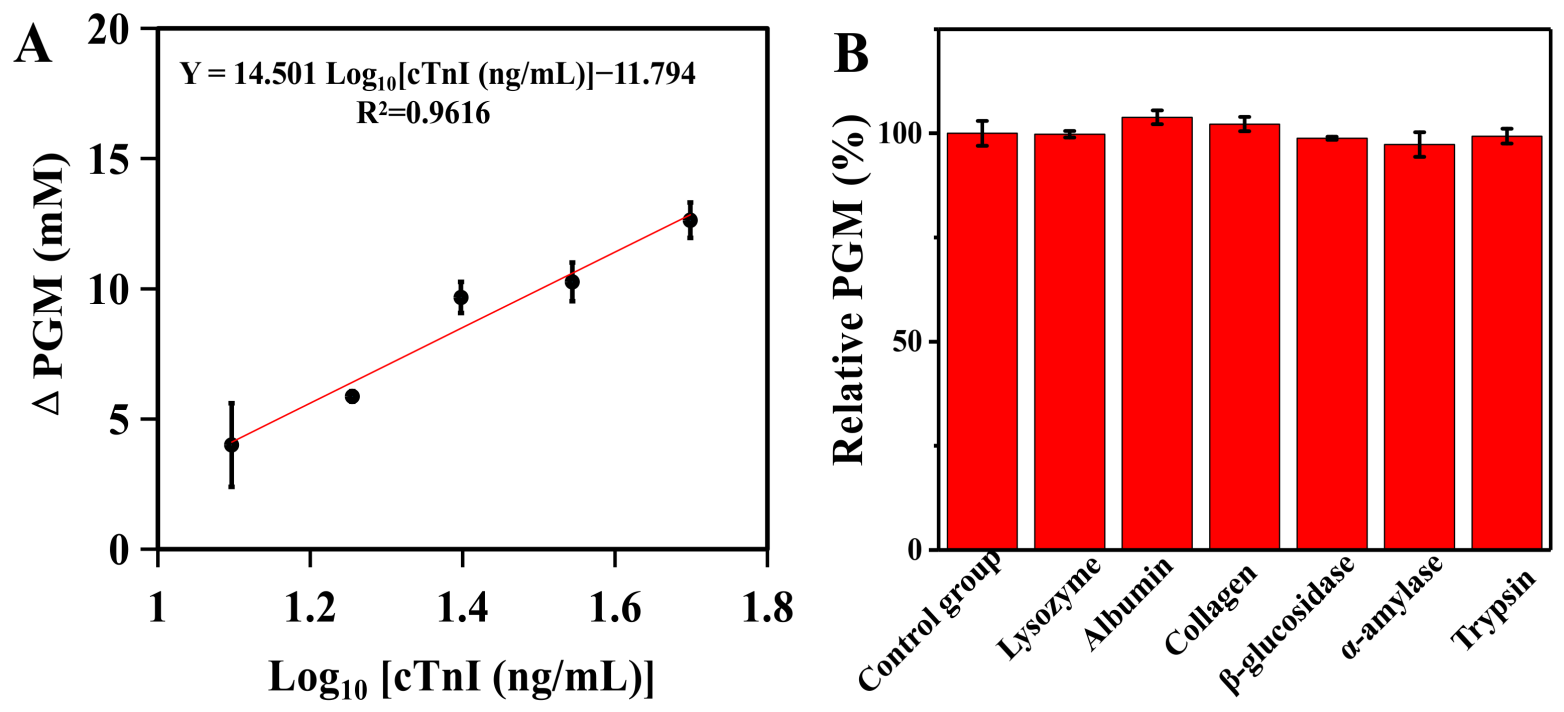
(**A**) Calibration plot for cTnI determination based on logarithmically transformed concentrations. The X-axis represents Log_10_ [cTnI (ng/mL)]. (**B**) Interference study of the cTnI assay.

Based on the broad use of HRP as an enzymatic label in ELISA, we further investigated whether the proposed HRP-responsive PGM system could be applied to HRP-labeled immunoassays for antigen detection. cTnI, a recognized indicator of acute myocardial infarction, served as the model analyte. The capture antibody was initially immobilized on a 96-well plate to bind cTnI. Then, the biotin-labeled detection antibody and HRP-labeled streptavidin were sequentially introduced through biotin-streptavidin recognition, allowing HRP to be retained on the plate. Finally, H_2_O_2_ was added, followed by TMB and DA, to generate a PGM-detectable signal related to the amount of cTnI. In Figure 7A, with increasing cTnI concentration, more HRP-labeled secondary antibodies were retained through the sandwich immunoreaction, leading to an enhanced ΔPGM response. The cTnI concentrations were logarithmically transformed before calibration. A linear dependence was obtained between ΔPGM and Log_10_[cTnI (ng/mL)] across the concentration range of 12.5–50 ng/mL. The calibration equation was Y = 14.501 × Log_10_ [cTnI (ng/mL)] − 11.794, with R^2^ = 0.9616. These findings confirm that the proposed PGM-based immunoassay enables quantitative cTnI detection within this concentration interval. The method based on PGM has a simpler reading format and does not require the use of specialized optical instruments. Moreover, this method eliminates the complex steps of signal marking preparation and can be directly operated using commercially available PGM and corresponding test strips. Considering the operational cost, portability, and convenience of detection, this method demonstrates significant practical advantages in the rapid detection of cTnI. To evaluate the possible interference caused by non-specific proteins during the cTnI detection process, lysozyme, albumin, collagen, β-glucosidase, α-amylase, and trypsin were selected as potential interfering substances. In Figure 7B, the relative PGM response values obtained after adding these proteins were very close to those of the control group, with only slight fluctuations. This indicates that these interfering substances have very little impact on the detection of cTnI, confirming that the proposed immunological analysis method based on the PGM has excellent anti-interference ability. To evaluate its performance in actual samples, a spiked recovery experiment was conducted, where cTnI was added to the serum samples; before analysis, the serum samples were diluted 1000 times. In Table 2, when the added concentration of cTnI was 15, 25, and 35 ng/mL, the recovery rate ranged from 93.0% to 117.1%, indicating that the developed portable glucometer immunological analysis method has a good application prospect for detecting cTnI in actual samples.

**Figure 8:**
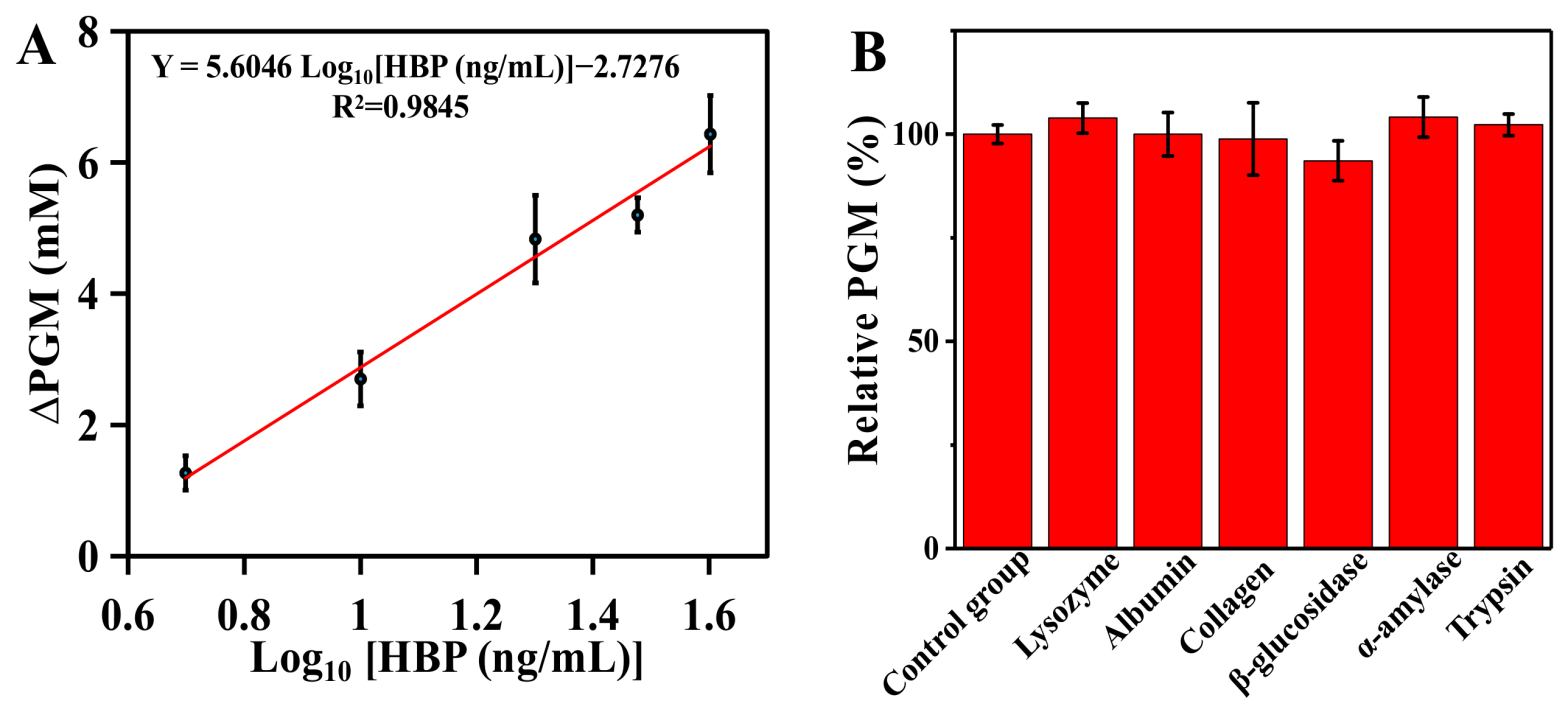
(**A**) Calibration plot for quantitative determination of HBP based on logarithmically transformed concentrations. The X-axis represents Log_10_ [HBP (ng/mL)]. (**B**) Interference study of the HBP assay.

In addition, the important biomarker of inflammation, HBP, was also selected as the target analyte for this study. A sandwich immunoassay system was constructed using this method. To achieve specific binding of HBP, the capture antibody was first immobilized on a 96-well plate; subsequently, after the antigen recognition was completed, the biotin-labeled detection antibody and HRP-labeled streptavidin were added successively, allowing HRP-labeled streptavidin to remain on the microplate. Subsequently, H_2_O_2_, TMB, and DA were added to trigger the substrate reaction and produce a PGM-readable signal associated with the HBP level. With increasing HBP concentration, more HRP-labeled streptavidin were retained through the sandwich immunoreaction, resulting in a gradually enhanced ΔPGM response. The HBP concentrations were logarithmically transformed before calibration. A linear dependence was obtained between ΔPGM and Log_10_[HBP(ng/mL)] across the concentration range of 5–40 ng/mL (Figure 8A). The calibration equation was Y = 5.6046 × Log_10_[HBP (ng/mL)] − 2.7276, with R^2^ = 0.9845. These results indicate that the proposed PGM-based immunoassay can respond effectively to HBP concentration changes within the tested range. To evaluate the anti-interference performance of the assay during HBP detection, lysozyme, albumin, collagen, β-glucosidase, α-amylase, and trypsin were selected as representative nonspecific proteins. In Figure 8B, the relative PGM responses after introducing these interferents remained close to that of the control group, with only slight variations. This result suggests that these proteins had negligible influence on HBP detection, confirming the good interference resistance of the proposed PGM-based immunoassay. To assess its performance in practical samples, serum samples spiked with HBP were subjected to recovery analysis. Before measurement, the serum samples were diluted 1000-fold and analyzed. As summarized in Table 2, when HBP was added at 10, 20, and 40 ng/mL, the recoveries ranged from 88.3% to 112.5%. These findings demonstrate that the portable PGM immunoassay can be applied to HBP determination in biological specimens.

### 3.5. Comparison with Previously Reported PGM-Based Immunoassays

Table 3 summarizes representative PGM-based immunoassay methods previously reported. Most prior studies have employed glucose oxidase-labeled markers, signal amplification strategies assisted by nanomaterials, nucleic acid amplification techniques, or electrochemical transduction interfaces. In contrast, this platform directly converts the catalytic activity of conventional HRP labels into a detectable response signal recognizable by PGM through the H_2_O_2_/TMB/DA reaction. This method is highly compatible with standard HRP-conjugated immunoassays and eliminates the need for glucose oxidase-labeled tags, nanomaterial-based amplification labels, nucleic acid amplification processes, or specialized electrochemical sensing interfaces. Although some PGM-based immunoassay methods have achieved lower LOD by introducing amplification strategies, these systems usually involve additional marker preparation and more cumbersome detection procedures. This method does not aim to surpass all platforms that rely on amplification in terms of analytical sensitivity, but rather focuses on directly converting the HRP labeling commonly used in traditional ELISA into portable digital signals. However, before achieving truly decentralized point-of-care testing, further integration of the immune reaction and washing steps is still a necessary prerequisite.

## 4. Conclusions

In this work, a portable immunoassay platform for HRP-related biomarker detection was developed based on a PGM. The proposed method employed HRP-mediated redox regulation of TMB and DA as the signal transduction strategy, enabling the conversion of HRP activity into a quantitative PGM readout. In the absence of HRP, TMB and DA reacted efficiently with [Fe(CN)_6_]^3−^ on the glucose test strip, producing a strong PGM response. In contrast, in the presence of HRP and H_2_O_2_, TMB was oxidized to ox-TMB, which further reacted with DA to generate oxidized products with weak reactivity toward [Fe(CN)_6_]^3−^, resulting in a decreased PGM signal. By integrating this reaction with an immunoassay format, target biomarker levels could be indirectly determined from HRP-dependent change in the PGM readout. This study broadens the use of PGM from conventional glucose monitoring to portable biomarker immunoanalysis. In addition, the spiked-serum recovery experiments provided a proof-of-concept demonstration of applicability. The main value of this strategy is the direct adaptation of conventional HRP-linked immunoassays to a commercial PGM readout without introducing an additional glucose-producing enzyme label. The current platform has lower throughput than automated microplate readers because PGM measurements are performed sequentially. Nevertheless, it is particularly suitable for individual or small-batch POCT, including applications in remote and resource-limited areas, where low cost, portability, simple operation, and minimal dependence on laboratory infrastructure are important. Although the platform is currently limited by a relatively narrow linear range, its advantage lies in directly converting traditional HRP labeling into signals recognizable by portable PGM, enabling quantitative analysis within validated ranges. Future research should focus on expanding the linear range and improving the signal conversion performance of the platform.

## Figures and Tables

**Table 1:** **Comparison of different methods for the detection of HRP.**

Materials	Detection Methods	Linear Range (ng/mL)	LOD (ng/mL)	Detection Time (min)	Readout Requirement	Portability ^a^	Ref.
Microfluidic paper-basedanalytical devices	Colorimetry	3–1000	5.58	>37.0	Optical/colorimetric measurement	Moderate	[23]
Methyl red	Electrochemistry	50–500	18	>10.0	Electrochemical workstation	Low	[24]
Poly beta-cyclodextrin	Electrochemistry	10–100,000	2.23	/	Electrochemical workstation	Low	[25]
ON/OFF-switchable magneto-controlled platform	Electrochemistry	0.1–200	0.097	/	Electrochemical workstation and magnetic nanomaterial	Low	[26]
Phenylboronic acid functionalized ionic liquid@ magnetic graphene oxide	Chemiluminescence	100–8000	29	60.0	Chemiluminescence reader and nanomaterial platform	Low	[27]
Multi-channel ink-jet ejection system	Chemiluminescence	10–1000	5	/	Automated ink-jet system and chemiluminescence instrument	Low	[28]
TMB/DA/H_2_O_2_	Electrochemistry (Personal glucometer)	156−625	26	5.0	Commercial personal glucose meter and disposable glucose test strip	High	This study

^a^ Portability was qualitatively assessed according to the need for laboratory-based instruments and specialized auxiliary devices. High: hand-held reader and disposable test strip; Moderate: simple optical or paper-based readout with auxiliary equipment; Low: dependence on an electrochemical workstation, chemiluminescence reader, or automated analytical system.

**Table 2:** Recovery study of cTnI and HBP in serum sample (*n* = 3).

Targets	Added (ng/mL)	Found ± SD (ng/mL)	Recovery (%)
cTnI	0	0	-
	15.0	13.9 ± 0.4	93.0
	25.0	29.3 ± 3.3	117.1
	35.0	33.7 ± 2.4	96.2
HBP	0	0	-
	10.0	8.8 ± 0.7	88.3
	20.0	20.1 ± 1.7	100.5
	40.0	45.0 ± 3.0	112.5

**Table 3:** Comparison of representative PGM-based immunoassays.

Targets	Signal-Conversion Strategy	Additional Component or Procedure	PGM Readout Principle	Practical Distinction from the Present Method	Ref.
Neuron-specific enolase	Glucoamylase- and antibody-functionalized gold nanoparticles	Glucoamylase tag and amylopectin hydrolysis	Glucose generated from amylopectin	Direct HRP-label conversion avoids an added glucose-producing enzyme tag	[15]
Neuron-specific enolase	Glucoamylase-labeled nanogold flowers	Nanogold flower synthesis, glucoamylase tag, and amylopectin hydrolysis	Glucose generated from amylopectin	No nanomaterial amplification tag or glucose-generating enzyme is required	[16]
Alpha-fetoprotein	Glucoamylase-labeled dendritic polyaniline nanofiber	Conducting-polymer nanofiber, glucoamylase tag, and amylopectin hydrolysis	Glucose generated from amylopectin	No nanofiber amplification material is required	[17]
Carbohydrate antigen 125	Invertase- and antibody-functionalized branched Pt nanowires	Pt nanowire synthesis, invertase tag, and sucrose hydrolysis	Glucose generated from sucrose	No Pt nanowire, invertase label, or sucrose-hydrolysis step is required	[18]
*Cronobacter sakazakii*	GOX-antibody silica nanoparticles and antibody-magnetic nanoparticles	Silica and magnetic nanoparticles; glucose oxidase-mediated glucose consumption	Decrease in residual glucose	No magnetic separation, glucose oxidase label, or nanoparticle pair is required	[19]
Alpha-fetoprotein	Rolling-cycle amplification with invertase-DNA conjugates	DNA amplification, enzyme-DNA conjugation, and sucrose hydrolysis	Glucose generated from sucrose	No nucleic-acid amplification or added glucose-producing reporter is required	[20]
Alpha-fetoprotein	Antibody-invertase cross-linkage nanoparticles	Invertase nanoparticles and sucrose hydrolysis	Glucose generated from sucrose	No invertase nanoparticle tag or sucrose-hydrolysis step is required	[21]
Carbohydrate antigen 19-9	Glucose oxidase-Cu-antibody immunoprobe	GOX-Cu hybrid nanoflower and glucose consumption	Decrease in residual glucose	No GOX-Cu nanozyme-like immunoprobe or glucose-consumption step is required	[22]
cTnI and HBP	HRP/H_2_O_2_/TMB/DA-mediated signal regulation	Conventional HRP-linked immunoassay and commercial glucose test strip	HRP-dependent modulation of the ferricyanide response on the test strip	Direct conversion of the conventional HRP label without an added glucose-producing enzyme or amplification tag	This work

## Data Availability

The original contributions presented in this study are included in the article. Further inquiries can be directed to the corresponding authors.
